# First Record and Identification of a Microsporidian Pathogen, *Nosema Maddoxi* in the Population of Brown Marmorated Stink Bug *Halyomorpha Halys* (Hemiptera: Pentatomidae) in Türkiye

**DOI:** 10.1007/s11686-024-00963-3

**Published:** 2025-01-24

**Authors:** Samet Eker, Mustafa Yaman, Ömer Ertürk, Tuğba Sağlam Güvendik, Gönül Algı

**Affiliations:** 1 Department of Molecular Biology and Genetics, Faculty of Arts and Science, Ordu University, Ordu, Türkiye; 2 Department of Biology, Faculty of Arts and Science, Bolu Abant İzzet Baysal University, 14030 Bolu, Türkiye

**Keywords:** *Halyomorpha halys*, *Nosema maddoxi*, Distribution, Türkiye, Biological control

## Abstract

**Purpose:**

The brown marmorated stink bug, *Halyomorpha halys* (Hemiptera: Pentatomidae), is an invasive and a highly polyphagous species with a strong dispersal capacity. Unfortunately, there is currently no effective control method that can prevent or reduce the economic loss caused by this pest. Among natural enemies, microsporidia cause infections in insects so that they can generally shorten life span, reduce fertility and inhibit growth.

**Methods:**

Current study involved field study, light and electron microscopy and molecular phylogenic analyses of a microsporidian pathogen in the populations of *H. halys* in Türkiye.

**Results:**

The microsporidian infection were mostly observed in malpighian tubules and fatty tissue filled with high spore density. Fresh uninucleate spores are oval, measued as 3.73 × 2.01 µm in dimensions. The mature spore wall is relatively thick and measures 75–85 nm and consists of a clear endospore (40–50 nm) and an electron-dense, uniform, thin exospore (25–30 nm). The polar filament is isofilar, 85–110 mm in diameter and has 7–8 coils. It is found to be most closely related to *Nosema maddoxi* isolate Mn.6 isolated from *H. halys* in Georgia in phylogenetic tree.

**Conclusion:**

The spore morphology and structural features of the isolate from *H. halys* identify it as *Nosema maddoxi*. The phylogenetic analyses confirm both light and electron microscopic observations. This is the first microsporidian record from *H. halys* and also from the order Hemiptera in Türkiye. It is also confirmed that the invasive pest, *H. halys* carries its natural pathogen, *N. maddoxi* to new geographical locations during its distribution.

## Introduction

The brown marmorated stink bug, *Halyomorpha halys* (Stål) (Hemiptera: Pentatomidae), is native to Asia (China, Japan, Korea and Taiwan) [1]. It was accidentally introduced to Europe and North America, where it has become a key pest, feeding on many important crops [2, 3]. It is considered a nuisance insect when it invades habitats as wintering grounds [4]. The pest, which has already spread to many countries including the USA, Canada, Chile, Germany, France, Spain, Italy, Greece, Austria, Hungary, Romania, Bulgaria, Slovakia, Serbia, Croatia, New Zealand, Russia, Georgia and Türkiye over a period of nearly 20 years [5], has expanded its distribution to the Caucasus and was first detected in Georgia in 2015 and established itself in 2016 with the first significant damage to hazelnuts being reported during 2016–2017 [6, 7]. It then spreads westwards to Türkiye and was detected and identified for the first time in Artvin province, Türkiye in 2017, which borders Batumi (Georgia), and started to continue its distribution in Türkiye [8, 9]. It is harmful to many types of fruits and vegetables [5, 6, 10–20]. It creates a risk of loss of yield and quality for many products by causing suction in fruits and vegetables as well as other hemipteran [21, 22]. Hazelnuts is critical host of the pest in Georgia and Türkiye [5–7]. Hazelnuts are one of the most important export products for Türkiye. It has been reported that the pest causes serious yield and quality losses in hazelnuts because it feeds directly on the fruit [5, 10, 11]. Today, various techniques are used to control pests, chemical insecticides or biological pest control using predators and parasitoids [7, 15, 23–28]. Insecticides provide short-term control of *H. halys* populations [25, 26], but increasing concerns about off-target effects, the development of resistance to insecticides and their toxicity to beneficial organisms mean that alternative control strategies need to be developed. *H. halys* is attacked or infected by various parasitoids, predators, and entomopathogens [23, 29]. Natural enemies associated with *H. halys* are being investigated in order to develop biological control strategies including entomopathogenic organisms [7, 27, 28]. Among entomopathogenic organisms, microsporidia can reach high prevalence in field populations of pest insects so that infection can negatively impact such field populations, especially when host populations are abundant [27]. Therefore, distribution of this microsporidian pathogen is of great importance to understand host–pathogen interaction and demostrate its natural supressor potential on the different geographic populations of the host. Here, we report the first record and identification of the microsporidian pathogen, *N. maddoxi* in *H. halys* populations in Türkiye.

## Materyal and Methods

### Insect Sample

In order to detect microsporidium pathogen in *H. halys* populations, adults and nymphs were collected from hazelnut orchards in October, 2023, Fatsa-Ordu, Türkiye. The samples were placed in sterile containers and brought to the laboratory and stored at 4 ^°^C.

### Light and Electron Microscopy

Firstly, each *H. halys* adult and nymph were carefully dissected in insect Ringer’s solution to preserve possible vegetative stages of the pathogen. A smear mount of body tissue was examined under the light microscope. When spores or developing stages of a microsporidian infection were detected in a wet smear, the slide was dried at room temperature. Air dried specimens were fixed in 100% methyl alcohol for 3–4 min, and stained with Giemsa-stain solution (Carloerba, No. 6B712176C) in a staining rack for one night. After that, the slides were washed in distilled water and air-dried. Giemsa-stained preparations were then carefully examined for the microsporidian infection [30, 31].

The ultrastructure of the pathogen was studied with a transmission electron microscope (TEM). Sample preparation for TEM was carried out according to standard techniques [31].

### Nucleic Acid Extraction, rRNA Gene Sequencing and Phylogenetic Analysis

Semi-purified spore suspensions were used for DNA extraction. In the first step of preparation for DNA extraction, heavily infected adults and nymphs were dissected individually under a stereo microscope. Infected tissues were removed from the insect body and collected in a 1.5 ml-Eppendorf tube where they were homogenized with Ringer’s solution using a micro pestle. The homogenates were filtered through three layers of muslin to remove gross insect debris. For DNA isolation, it was started by agitating the microsporidian spores with glass beads as offered by Hyliš et al. [32]. Then, the procedures were folowed with the DNA isolation kit (QIAGEN DNA Isolation Kit-69504, Hilden, Germany), according to Yaman et al. [31] and the manufacturer’s guidelines. To amplify the microsporidian SSU rRNA genes, the 18F/1537R primer sets (18F/1537R: 5’-CACCA GGTTG ATTCT GCC-3’/5’- TTATG ATCCT GCTAA TGGTT C-3’) and multiplex PCR kit (QIAGEN Multiplex-206143) were used. The amplification was performed according to Yaman et al. [31]. The PCR-amplified products were loaded onto a 0.9% agarose gel, which was supplemented with ethidium bromide. The PCR products and the primers used for PCR were then sent to the Macrogen Inc. Company, Netherlands for determination of the base sequences.

The GenBank accession numbers of microsporidian SSU rRNA gene sequences from 28 microsporidians used in the phylogenetic analysis are listed in Table 1. SSU rRNA gene sequences (Table 1) were aligned with the maximum likelihood method using Kimura two-parameter distance and evaluated by 1000 bootstrap replications with the MEGA.X program. The 28 SSU rRNA sequences belonging to various microsporidia species that produced the highest scores in the BLAST search were included in the analysis. The GC content of the SSU rRNA sequence of the microsporidium were analyzed with the %G ~ C Content Calculator Program with web design.

**Table 1:** Species and accession numbers selected from the gene bank (ncbi) to be used in phylogenetic analysis

Organism name	Accession number
*Nosema maddoxi*	MT434671
*Nosema maddoxi* isolate Nm6	MT434673
*Nosema maddoxi* isolate Nm3	MT434672
*Nosema maddoxi* isolate Nm1	MT434670
*Vairimorpha cheracis*	AF327408
*Nosema sp.* isolate L105	MK241529
*Nosema granulusis*	AJ011833
*Vairimorpha austropotomobi*	MF344634
*Nosema granulosis* partial ssu gene	FN434088
*Nosema* sp*.*	GQ337009
*Nosema* sp GKK-2009	GQ337703
*Microsporidium* sp.	KY615712
*Nosema* sp. gene for small subunitribozomal RNA	AB009977
*Nosema mylitta*	MN542655
*Vairimorpha imperfecta* 16srRNA gene	AJ131645
*Vairimorpha imperfecta*	AJ131646
*Nosema fumiferaae* internal transcribed spacer and 16S	KT020736
*Nosema bombycis*	MT510137
*Nosema* sp. 22KM-1	LC722845
*Nosema* sp*. YXu-*2022a	OP821984
*Nosema* sp*. OSL-2012-5*	LC052197
*Nosema trichoplusiae*	U09282
*Nosema* sp*. S. litura*	AF238239
*Nosema bombycis* gene for small subunit rRNA	D85504
*Nosema* sp*.* TWSL-2014-2	LC422304
*Nosema pyrausta*	AY958071
*Nosema bombycis* strain GNBg	MT510134
*Nosema* sp*. TWSL-2014-1*	LC422303
*Tryponosoma congolense*	FN265929
*Tryponosoma congolense* isolate IL300	FN265926
*Nosema maddoxi* TR	-

## Results

In the present study, a microsporidian pathogen from the populations of *H. halys* in Türkiye was investigated using light and electron microscopy and molecular phylogenic analyses. The pathogen was found in that population in Ordu, Türkiye for the first time in 2023. Nineteen of the 154 *H. halys* adults examined in October 2023 were infected by the microsporidium. The total infection rate was 12.3%.

### Microscopy

**Fig. 1:**
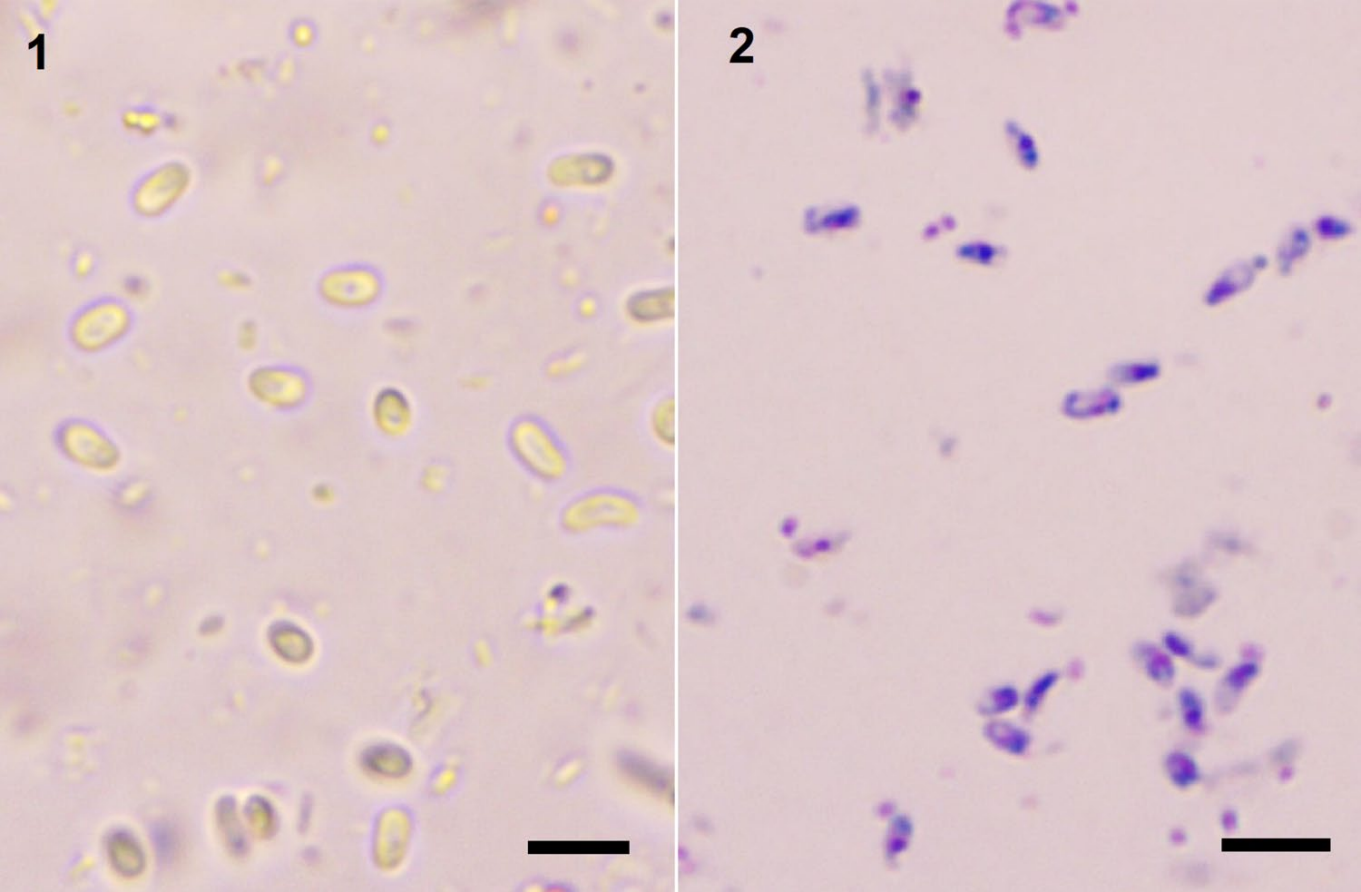
Light microscopy micrographs of *Nosema maddoxi* in *Halyomorpha halys*. 1-Fresh spores, 2-Giemsa-stained spores. Scale bar 10 mm in both figures

**Fig. 2:**
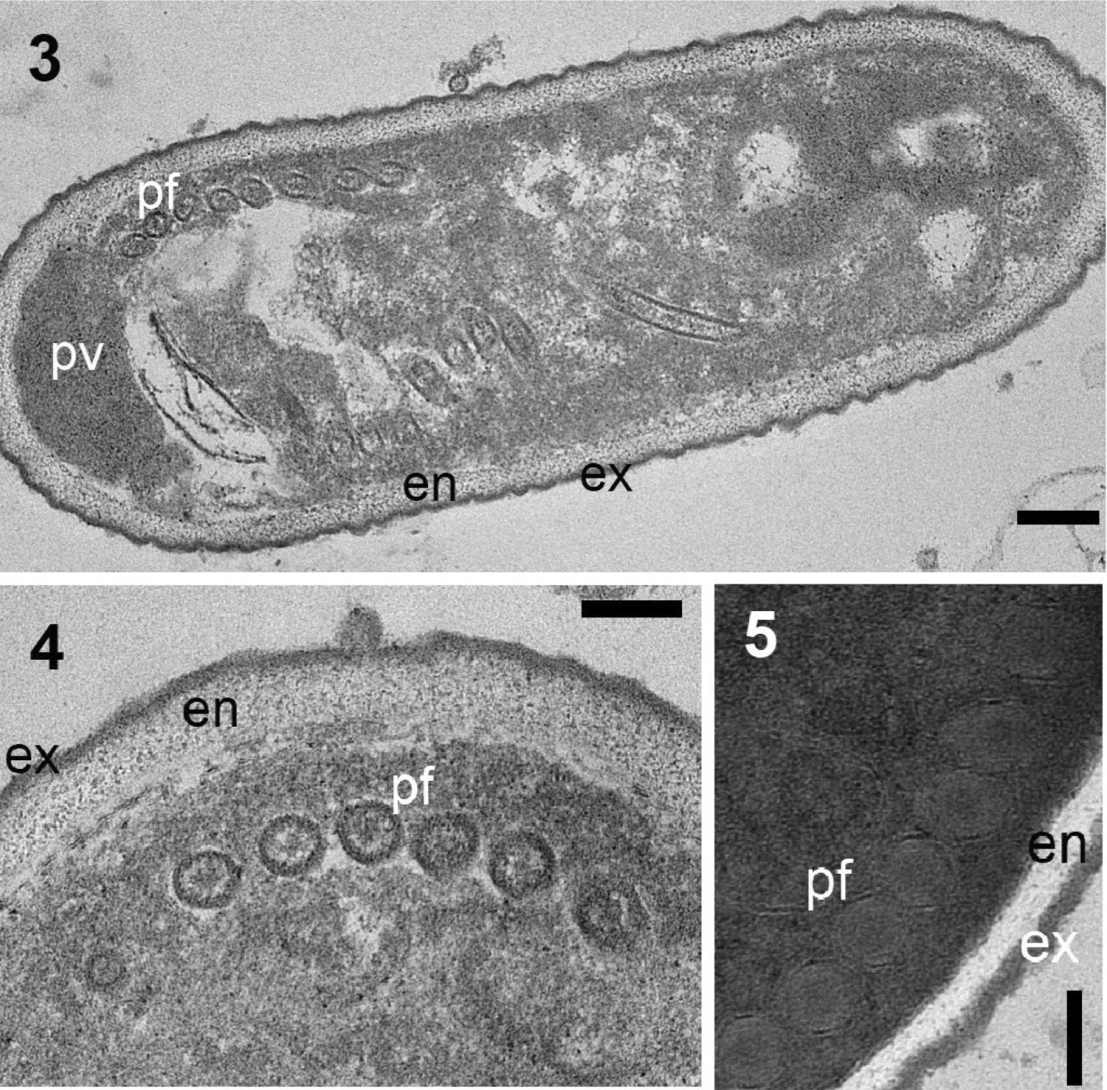
Transmission electron micrographs of *Nosema maddoxi* from *Halyomorpha halys*. 3-Longitudinal section of a spore, showing seven coils of the polar filament, 4–5-Cross section of the isofilar polar filament with 7 coils. (pf), exospore (ex) and endospore (en). Scale bar 200 mm in Figure 3, 100 nm in Figures 4 and 5

First light microscopic study revealed that the infection were mostly observed in malpighian tubules and fatty tissue filled with high spore density. Fresh uninucleate spores are oval, measued as 3.73 ± 0.18 µm (from 3.18 to 4.36 µm) (n = 30) in length and 2.01 ± 0.28 µm (from 1.62 to 2.35 µm) (n = 30) in width (Fig. 1). The Giemsa stained spores were 3.46 ± 0.29 µm (from 3.0 to 4.17 µm) (n = 50) in length and 1.71 ± 0.16 µm (from 1.37 to 2.06 µm) (n = 30) in width (Fig. 1). Light and electron microscopic observations confirmed that the developmental stages and spores were in direct contact to the host cell cytoplasm (Figs. 1, 2). A sporophorous vesicle was not observed during the light and electron microscopical observations. Ultrastructural studies showed that the free, oval spores contain polar flaments and relatively tick spore wall consisted of endospore and exospore, typicals for microsporidia. The mature spore wall is relatively thick and measures 75 to 85 nm and consists of a clear endospore (40–50 nm) and an electron-dense, uniform, thin exospore (25–30 nm) (Fig. 2). The spore has a large posterior vacuole filled with the remaining material from the polar filament formation. The polar filament is isofilar, 85–110 mm in diameter and has 7–8 coils (Fig. 2).

### Phylogenetic Analysis

**Fig. 3:**
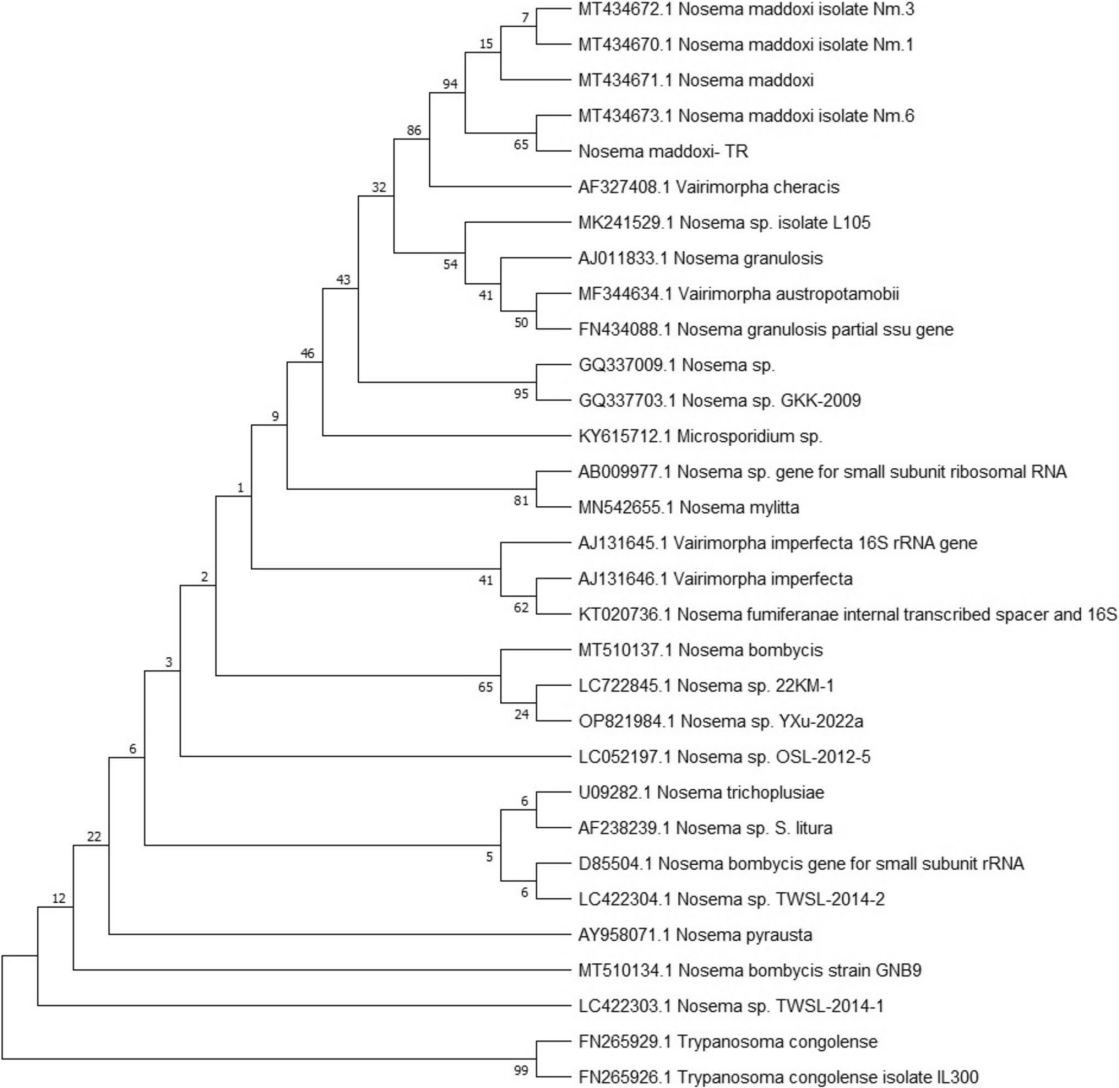
Phylogenetic relationships of the microsporidian isolate from *H. halys* among microsporidium species isolated from different hosts based on SSUrRNA. The tree was constructed by maximum likelihood method using Kimura two-parameter distance and evaluated by 1000 bootstrap replications with the MEGA.X program. *Trypanosoma congolense* and *Trypanosoma congolense* isolate L300 were used as outgroups in the analysis

The PCR-amplified fragment of the SSU rRNA gene of the microsporidum was successfully sequenced. A NCBI-BLAST search revealed similarities with the sequences of *Nosema* species. A phylogenetic tree was constructed by including 28 SSU rRNA sequences belonging to *Nosema* and *Vairimorpha* species that produced the highest scores in the BLAST search in the analysis (Table 1, Fig. 3). The phylogenetic tree produced several clades including *Nosema* and *Vairimorpha* species, based on the host insect species. Our isolate clustered with the *Nosema* group including *N. maddoxi* species (Fig. 3). It is found to be most closely related to *Nosema maddoxi* isolate Mn.6 isolated from *H. halys* in Georgia. Analysis of length and GC content of the SSU rRNA gene of *N. maddoxi* from this study was also done. 1186 bps were used in the analyses, we found a GC content of the microsporidian base sequences of 33.3%.

## Discussion

Light, electron microscopy and molecular phylogenic analyses confirm that the pathogen found in *H. halys* population is a microsporidium. The ultrastructural characteristics of the spore are important features for confirming of microsporidian species. The ultrastructure of the spore elucidated the typical characteristics of microsporidia such as a polar filament, a posterior vacuole, a lack of mitochondria and tick spore wall consisted of endospore and exospore (Fig. 2) [33–35]. Considering the giemsa staining spore morphology and spore ultrastructural characteristics of the microsporidial pathogen of *H. halys,* we conclude that it belongs to the genus *Nosema* Naegeli, 1857. The microsporidian pathogen recorded in this study has the typical characters of the genus *Nosema* such as absence of sporophorous vesicles, uniform exospore and the thickness of the spore wall [33–35].

To date, the sole finding of a microsporidium infecting *H. halys* was *Nosema maddoxi* [7, 8, 29] and no later record of any other diplokaryotic microsporidium species from *H. halys* has been recorded. Here we present a microsporidium record, *Nosema maddoxi* from *H. halys* for the first time in its populations in Türkiye. The tissue specificity of the microsporidian isolate from *H. halys* in Türkiye is similar to that described in the literature [6, 7, 29]. On the other hand, the spore dimension is a suitable morphological character to compare pathogens or their different geographic isolates. The microsporidian from *H. halys* in this study differs from the original description of *N. maddoxi* in spore dimensions. Our isolate has smaller spores (3.73 ± 0.18 × 2.01 ± 0.28 µm) than in the original description (4.72 ± 0.05 × 2.19 ± 0.03 µm) of *N. maddoxi* [29]. However, the microsporidium in this study shows similarities with *N. maddoxi* from *H. halys* in Georgia*,* neighboring country to Türkiye, concerning spore size (3.76 ± 0.56 × 1.72 ± 0.28 µm) and infected host species and tissue (fat body and Malpigian tubules), as described by Kereselidze et al. [6, 7, 29]. Our microsporidian also shows similarity with the original description of *N. maddoxi* in the number of polar coils, providing a further useful taxonomic criterion for differentiating species [35]. The number of polar coils of our microsporidian isolate (7–8) is similar to that of the original description of *N. maddoxi* in USA [29]. The spore morphology and structural features of our microsporidian isolate from *H. halys* identify it as *N. maddoxi*. The phylogenetic analyses confirm both light and electron microscopic observations. According to the phylogenetic tree, our isolate is found to be most closely related to *Nosema maddoxi* isolate Mn.6 isolated from *H. halys* in Georgia (Fig. 3). The microsporidian pathogen, *Nosema maddoxi* was identified as infecting *H. halys* in the USA for the first time [29]. This microsporidium was also found to infect *H. halis* in China and South Korea [29]. Studies in the USA found *N. maddoxi* to infect *H. halys* in every state sampled [36]. And than *N. maddoxi* was reported to infect *H. halys* in three sites in the Guria region of western Georgia in 2018 [7]. Up to now, no microsporidium record from *H. halys,* furthermore from the order Hemiptera in Türkiye. This is the first microsporidian record from *H. halys* and also from the order Hemiptera in Türkiye.

*H. halys* is an invasive pentatomid introduced from Asia into the United States, Canada and European countries and a highly polyphagous species with a strong dispersal capacity and high reproductive output [3, 5, 13, 14, 37, 38]. Later, this pest was first reported attacking hazelnut orchards in the Republic of Georgia in 2015 [7] and then in İstanbul [39] and later Artvin [8, 9], Türkiye in 2017. *H. halys,* which entered Türkiye from the Georgian border region [8, 9] and spread to hazelnut production areas throughout the Black Sea region [5], is the most important pest of hazelnut areas in Türkiye and the only cause of irreparable economic losses. Unfortunately, there is currently no effective control method that can prevent or reduce the economic loss caused by this pest in hazelnut areas. Laboratory bioassays show that *H. halys* infected by this pathogen exhibit reduced fertility and longevity [27]. On the other hand, Microsporidia cause infections in insects so that they generally can shorten life spans, reduce fertility and inhibit growth. Furthermore, as an entomopathogenic group, microsporidia can also reach high prevalence in field populations of pest insects so that infection can negatively impact such field populations, especially when host populations are abundant [27]. Therefore, distribution of this microsporidian pathogen is of great importance to understand host–pathogen interaction and demostrate its natural supressor potential on the different geographic populations of the host. Determining the presence of any microsporidian pathogen in natural populations as a natural suppressive factor in *H. halys* populations, which rapidly spreads and causes damage, will contribute to integrated pest management (IPM) against this pest. In this study, while the first record of microsporidium, *N. maddoxi*, the natural pathogen of *H. halys,* is given in Türkiye for the first time, it is also confirmed that the invasive pest, *H. halys* carries its natural pathogen, *N. maddoxi* to new geographical locations during its distribution.

## Data Availability

The data that support the findings of this study are available from the first author upon reasonable request.
